# Early major adverse kidney events after lung transplantation: risk of chronic kidney disease and prognostic impact

**DOI:** 10.1007/s11748-025-02193-4

**Published:** 2025-08-28

**Authors:** Yoshihisa Shimada, Chihiro Konoeda, Yue Cong, Masaaki Nagano, Keita Nakao, Mitsuaki Kawashima, Akinori Maeda, Kent Doi, Norihiko Ikeda, Masaaki Sato

**Affiliations:** 1 Department of Thoracic Surgery, The University of Tokyo Graduate School of Medicine, 7-3-1 Hongo, Bunkyo-ku, Tokyo 113-0033 Japan; 2 Department of Thoracic Surgery, Tokyo Medical University, Tokyo, Japan; 3 Department of Emergency and Critical Care Medicine, The University of Tokyo Graduate School of Medicine, Tokyo, Japan

**Keywords:** Lung transplantation, Acute kidney injury, Chronic kidney disease, Prognosis

## Abstract

**Objective:**

Renal dysfunction, including acute kidney injury (AKI) and chronic kidney disease (CKD), is a major complication following lung transplantation (LT) and is associated with increased morbidity and mortality. This study aims to evaluate the clinical significance of AKI in relation to the development of post-LT CKD and poor prognosis.

**Methods:**

Among 133 patients who underwent LT, 116 were included in the analysis. AKI was defined according to the Kidney Disease Improving Global Outcomes criteria. AKI was classified into early AKI (E-AKI), occurring within a few hours to one week postoperatively, and late AKI (L-AKI), occurring between one week and one month after LT. The Major Adverse Kidney Event within 30 days following LT (MAKE30)—a composite outcome that includes all-cause mortality, new renal replacement therapy, or persistent renal dysfunction—was also used in this study. Univariate and multivariate analyses were conducted to identify factors associated with the development of CKD. Overall survival (OS) was analyzed using the Kaplan–Meier method.

**Results:**

The proportions of patients who developed E-AKI, L-AKI, MAKE30, and CKD were 73%, 31%, 15%, and 46%, respectively. Multivariate analysis identified older age and the occurrence of MAKE30 as independent predictors of post-LT CKD. Notably, all recipients aged 50 years or older who experienced either L-AKI or MAKE30 subsequently developed CKD. In addition, the incidence of MAKE30 was marginally correlated with reduced OS.

**Conclusion:**

The occurrence of L-AKI and MAKE30 following LT is associated with the development of CKD and MAKE30 also has a negative impact on OS.

**Supplementary Information:**

The online version contains supplementary material available at 10.1007/s11748-025-02193-4.

## Introduction

Lung transplantation (LT) is a treatment option for patients with end-stage respiratory failure. The evolution of transplantation techniques has led to an increase in the median survival post-LT, benchmarked at 6.7 years [1]. However, when juxtaposed with other solid organ transplants, the long-term outcomes post-LT lag behind. A lot of factors have been identified that affect both short- and long-term outcomes after LT [1, 2]. Among these, renal dysfunction, which includes conditions, such as chronic kidney disease (CKD) and acute kidney injury (AKI), is one of the most prevalent complications [2–10].

CKD after LT is associated with high morbidity, mortality, and cost [2, 8]. The need for renal replacement therapy (RRT) shortly after LT elevates the adjusted hazard ratio for 1-year and 5-year mortality to 7.2 and 4.0, respectively [2]. Identifying patients at high risk of developing advanced renal failure is crucial as it would enable timely therapeutic intervention.

AKI is a complex syndrome characterized by a decrease in the glomerular filtration rate (GFR), leading to elevated serum creatinine (sCr) levels and a decline in urine output over a specific time interval. It is associated with higher morbidity, mortality, and primary graft dysfunction, with incidence rates following LT ranging from 9.4% to 69% [5, 11]. Several classification systems are used in the current practice to assess AKI severity, among which the Kidney Disease Improving Global Outcomes (KDIGO) criterion is one of the most widely accepted consensus definitions [12].

In contrast, the Major Adverse Kidney Event (MAKE)—a composite outcome that includes all-cause mortality, new RRT, or persistent renal dysfunction—is a well-recognized endpoint, and MAKE within 30 days (MAKE30) has emerged as a valid measure in AKI clinical trials [13–15]. However, to date, no studies have simultaneously applied both the KDIGO criteria and the MAKE30 to predict the incidence of CKD following LT.

Few of these classification systems consider the timing or duration of AKI. Kim et al. conducted the only study analyzing AKI characteristics after LT based on the timing of onset [16]. They defined early AKI (E-AKI) as occurring within one week after LT, and late AKI (L-AKI) as occurring between one week to one month after LT. Their findings indicated that E-AKI presented with a more advanced AKI stage and a higher proportion of patients requiring RRT due to renal failure compared to L-AKI [16]. Nevertheless, the clinical significance of AKI timing, particularly its impact on long-term survival and the development of CKD after LT remains unclear.

Given that the considerable physical stress and difficulty in maintaining hemodynamic stability soon after LT, evaluating AKI without considering the timing of its occurrence may not accurately reflect the overall trajectory of renal function decline. This study aimed to investigate the clinical significance of AKI timing based on the KDIGO, and to identify clinico-surgical factors including MAKE 30 associated with the development of post-LT CKD.

## Patients and methods

### Patients

**Fig. 1:**
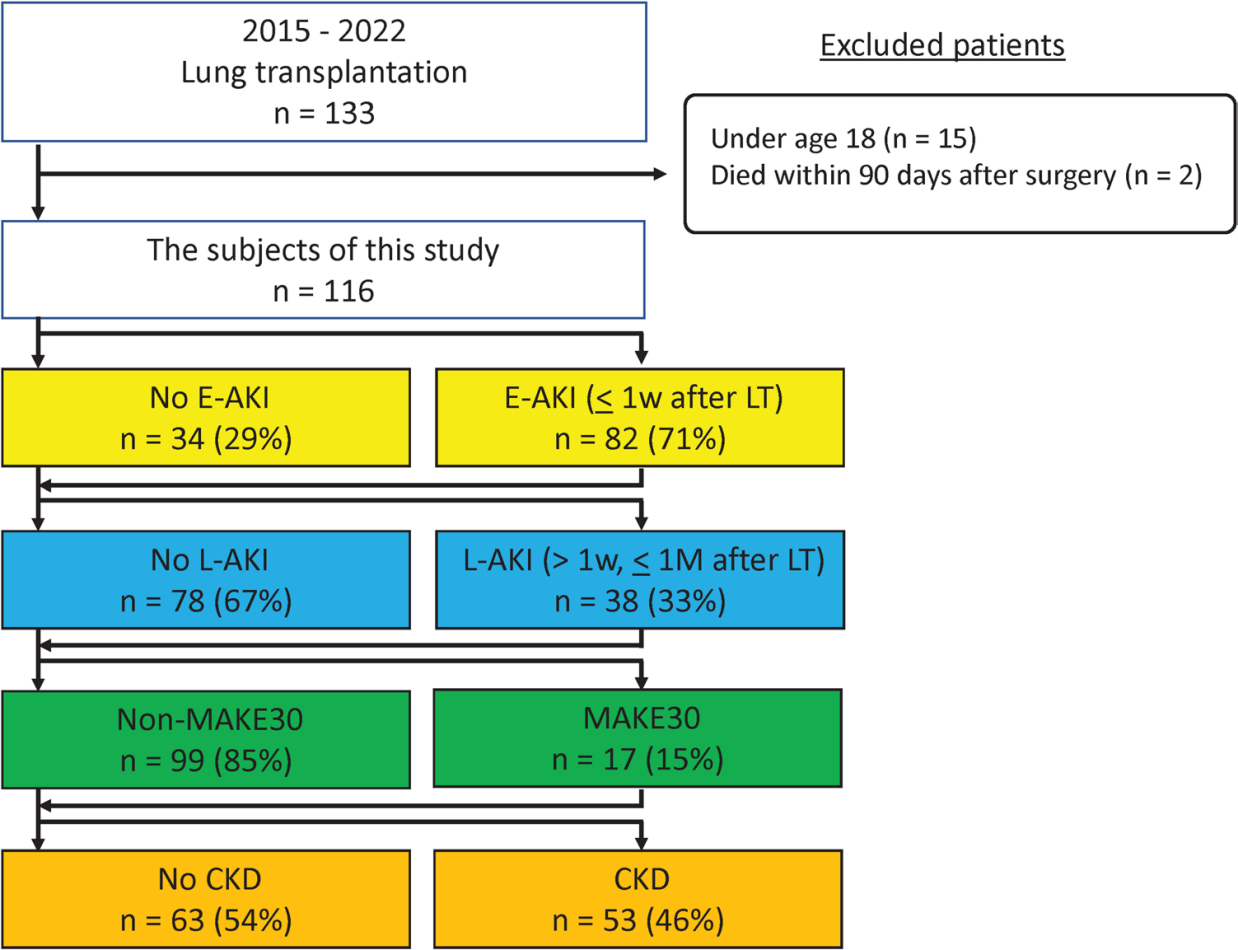
Consort diagram of patients included in the study. *E-AKI* early acute kidney injury; *L-AKI* late acute kidney injury; *MAKE30* Major Adverse Kidney Event within 30 days; *CKD* chronic kidney disease

Between January 2015 and December 2022, 133 patients underwent LT. We applied the following exclusion criteria: age < 18 years and death within 90 days post-LT. Thus, 116 patients were included in the study. A consort diagram detailing patient inclusion is shown in Fig. 1.

All patients underwent triple maintenance immunosuppression with mycophenolate mofetil, steroids, and calcineurin inhibitors, such as tacrolimus and cyclosporine. Induction therapies such as basiliximab are not routinely used. Post-Lt, the target tacrolimus levels were set at 12–15 ng/mL for the initial 3 months, 10–12 ng/mL from the 3 to 6 months, and 8–12 ng/mL thereafter. However, these targets may be modified based on patient-specific factors, such as age, body weight and size, and organ function. m-TOR inhibitors, including sirolimus and everolimus, were not used for immunosuppression because they were not covered by the Japanese National Health Insurance Program. We estimated the variability in absolute blood tacrolimus blood levels using the coefficient of variation (CV), which was calculated according to the following equation: [17, 18].

CV (%) = standard deviation/mean tacrolimus concentration x 100

During LT, we monitored pulmonary artery pressure, cardiac output, and arterial blood gas levels. Cardiopulmonary bypass (CPB) or extracorporeal membrane oxygenation (ECMO) was used in patients with severe pulmonary hypertension and/or right ventricular dysfunction. Additionally, it was selectively used in patients who exhibited instability during the transplantation process. Postoperative hemodynamic monitoring was performed. Aggressive diuretics were administered to ensure a negative fluid balance. In our facility, during the refilling phase, continuous intravenous infusion of furosemide should be actively employed to prevent heart failure and pulmonary edema. If the response to furosemide is insufficient, low-dose carperitide may be administered given its diuretic and inotropic effects, as well as its potential renoprotective properties. Prophylactic antibiotics were administered peri-operatively. All the procedures performed involving human participants were performed in accordance with the Declaration of Helsinki (2013). This study was approved by the ethics committee of The University of Tokyo Graduate School of Medicine (clinical pilot study no.2406).

### Definition of E-AKI, L-AKI, MAKE30, and CKD in this study

Preoperative renal function data include sCr levels and estimated GFR (eGFR). We used the KDIGO criteria to define AKI. E-AKI could be identified by any of the following indicators: an increase in sCr by 0.3mg/dL or more within 48 h post-LT; an increase in sCr to 1.5 times the baseline or more within the first 7 days post-LT; or a urine output of less than 0.5 mL/kg/h for 6 h. L-AKI was defined as AKI that emerged after the first week but within one month post-LT. MAKE30 is a composite outcome comprising all-cause mortality, initiation of new RRT, or persistent renal dysfunction within 30 days after LT, defined as a postoperative serum creatinine level before hospital discharge greater than twice the preoperative baseline value. The criteria for postoperative CKD were defined as patients having an eGFR ≤ 60 mL/min/1.73m^2^ for a period exceeding 3 months post-LT. Accordingly, patients who died within 90 days post-LT were excluded from the analysis. The definitions of E-AKI, L-AKI, MAKE30, and CKD are summarized in Table 1.

**Table 1:** Definitions of acute kidney injury, major adverse kidney event within 30 days, and chronic kidney disease in this study

Status	Stage I	Stage II	Stage III
E-AKI (Within 1 week following LT)	1. sCr increase > 0.3 mg/dL within 48 h following LT 2. sCr increase x 1.5–1.9 3. Urine output < 0.5 mL/kg/h for over 6 h	1. sCr increase x 2.0–2.9 2. Urine output < 0.5 mL/kg/h for over 12 h	1. sCr increase x 3.0 2. sCr ≥ 4 mg/dL 3. initiation of RRT 4. Urine output < 0.3 mL/kg/h for over 24 h
L-AKI (> 1 week, ≤ 1 month following LT)	1. sCr increase x 1.5–1.9 2. Urine output < 0.5 mL/kg/h for over 6 h		
MAKE30	One or more of the following criteria met in the 30 days after LT (no stages) 1. In-hospital mortality: death prior to hospital discharge 2. New receipt of RRT after LT: Receipt of any modality of RRT prior to hospital discharge in a patient not known to have previously receipt RRT. 3. Persistent renal dysfunction: postoperative sCr before hospital discharge ≥ x 2.0 of the preoperative sCr		
Chronic kidney disease	An eGFR < 60 ml/min/1.73 m^2^ for three months or more following LT (no stages)		

*E-AKI* early-acute kidney injury; *LT* lung transplantation; *L-AKI* late-acute kidney injury; *MAKE30* major adverse kidney event within 30 days; *sCr* serum creatinine level; *RRT* renal replacement therapy; *eGFR* estimated glomerular filtration rate

### Statistical analysis

Overall survival (OS) was measured from the day of LT until the day of death from any cause or the last known date the patient was alive. Survival curves were plotted using the Kaplan–Meier method. Differences in variables were determined using the log-rank test. Univariate and multivariate analyses were performed to identify factors associated with the incidence of CKD following LT. Pearson’s chi-square test for categorical data and Student’s t test for continuous data were used for univariate analyses. A forced entry method was used to build the logistic regression models. A receiver operating characteristics (ROC) curve of a significant numerical factor for CKD was constructed, and the optimal cut-off value was determined using the area under the curve (AUC). All tests were two-sided, and *P* values of less than.05 were considered to indicate statistical significance. The IBM SPSS Statistics 28.0 software package (DDR3 RDIMM; IBM Corp, Armonk, NY, USA) was utilized for statistical analysis.

## Results

The characteristics of the 116 recipients based on the incidence of CKD after LT are shown in Table 2. Of these, 53 (45.7%) developed CKD. The proportions of patients who experienced E-AKI, L-AKI, and MAKE30 were 71% (*n* = 82), 33% (*n* = 38), and 15% (*n* = 17), respectively. The numbers of patients with stage I, II, and III E-AKI were 41 (35%), 38 (33%), and 3 (3%), respectively. For L-AKI, the numbers of patients with stage I, II, and III disease were 24 (21%), 7 (6%), and 7 (6%), respectively. There were no significant differences in background characteristics between the CKD and non-CKD patients, except for age (*P* =.038). CKD patients had preoperative lower serum hemoglobin (*P* =.005) and albumin (*P* =.007), and increased ECMO use (*P* =.026). CKD patients had a longer circulatory support time (*P* =.007) and operation time (*P* <.001), while they had a higher likelihood of requiring RRT (*P* =.014), postoperatively. CKD patients exhibited elevated sCr levels and reduced eGFR levels during the postoperative acute (sCr *P* =.023, eGFR *P* =.003) and sub-acute period (sCr *P* =.021, eGFR *P* <.001). The incidences of L-AKI (*P* <.001) and MAKE30 (*P* =.001) were significantly associated with an increase in CKD, whereas that of E-AKI (*P* =.530) was not.

**Table 2:** Characteristics of 116 recipients according to chronic kidney disease following lung transplantation

Variables, mean ± SD	CKD after LT + *n* = 53	CKD after LT - *n* = 63	*P* value
Age, years	48 ± 11	44 ± 13	0.038
Sex, male (%)	25 (47)	38 (60)	0.157
Any smoking history (%)	18 (34)	28 (44)	0.250
Brain-dead donor (%)	46 (87)	54 (86)	0.867
Comorbidities (%)	27 (51)	30 (48)	0.721
Hypertension	2	3	0.794
Diabetes mellitus	2	2	0.860
Underlying diseases (%)			0.709
Interstitial lung disease	21 (40)	24 (38)	
COPD	2 (4)	5 (8)	
Pulmonary hypertension	7 (13)	4 (6)	
GVHD	6 (11)	6 (10)	
*Preoperative factors*			
Hb, g/dL	12.59 ± 2.30	13.51 ± 2.10	0.005
Cr, mg/dL	0.62 ± 0.19	0.64 ± 0.21	0.397
eGFR	103.07 ± 32.69	108.70 ± 38.40	0.196
Albumin, g/dL	3.83 ± 0.52	4.03 ± 0.51	0.007
Steroid use (%)	29 (55)	27 (43)	0.203
ECMO use (%)	4 (8)	0	0.026
*Intraoperative factors*			
Circulatory support (%)	37 (70)	45 (71)	0.849
Circulatory support time, min	389.18 ± 320.07	212.61 ± 263.68	0.042
Bilateral transplant (%)	26 (49)	36 (57)	0.287
Operation time, min	516.78 ± 345.20	305.66 ± 324.95	< 0.001
Transfusion volume, mL	5822.58 ± 7234.80	4077.98 ± 5660.94	0.084
Blood loss, mL	5992.74 ± 10530.68	3513.75 ± 6120.77	0.065
Ischemic time, min	429.01 ± 238.26	379.39 ± 223.50	0.125
*Postoperative factors*			
Use of ECMO (%)	11 (21)	16 (25)	0.556
Antibiotics use, VCM or AG (%)	16 (30)	18 (29)	0.849
Sepsis (%)	3 (6)	2 (3)	0.511
Acute rejection (%)	12 (23)	13 (21)	0.793
CV of tacrolimus in the acute phase, %	30.9 ± 12.1	29.9 ± 10.2	0.327
CV of tacrolimus in the late acute phase, %	24.3 ± 8.6	24.8 ± 9.2	0.440
Renal replacement therapy (%)	7 (13)	1 (2)	0.014
Cr (≤ 1 week), mg/dL	0.95 ± 0.48	0.79 ± 0.27	0.023
eGFR (≤ 1 week)	70.59 ± 28.82	86.21 ± 31.00	0.003
E-AKI (≤ 1 week, %)	39 (73)	43 (68)	0.530
Stage 1	18 (34)	23 (37)	
Stage 2	18 (34)	20 (32)	
Stage 3	3 (6)	0	
Cr (> 1 week, ≤ 1 month), mg/dL	0.97 ± 0.69	0.75 ± 0.50	0.021
eGFR (> 1 week, ≤ 1 month)	76.08 ± 34.96	99.46 ± 40.49	< 0.001
L-AKI (> 1 week, ≤ 1 month, %)	26 (49)	12 (19)	< 0.001
Stage 1	16 (30)	8 (13)	
Stage 2	5 (9)	2 (3)	
Stage 3	5 (9)	2 (3)	
MAKE30	14 (26)	3 (5)	0.001

*CKD* chronic kidney disease; *LT* lung transplantation; *SD* standard deviation; *COPD* chronic obstructive pulmonary disease; *GVHD* graft-versus-host disease; *Hb* hemoglobin; *Cr* creatinine; *eGFR* estimated glomerular filtration rate; *VCM* vancomycin; *AG* aminoglycoside antibiotics; *CV* coefficient of variation; *E-AKI* early-acute kidney injury; *L-AKI* late-acute kidney injury; *MAKE30* Major Adverse Kidney Events within 30 days after lung transplant

**Fig. 2:**
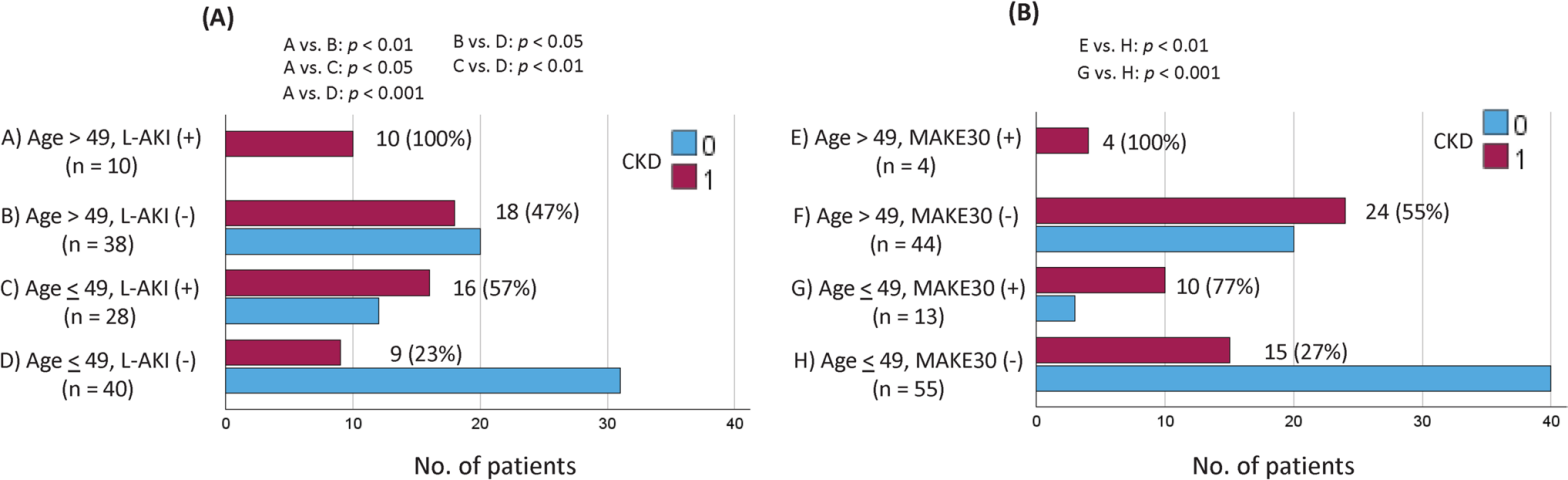
**A** Proportions of chronic kidney disease (CKD) in patients after lung transplantation (LT), considering the combined influence of two risk factors, L-AKI and age. **B** Proportions of CKD in patients after LT, considering the combined influence of two risk factors, MAKE30 and age. *L-AKI* late acute kidney injury; *MAKE30* Major Adverse Kidney Event within 30 days; *CKD* chronic kidney disease

The results of the multivariate analysis for CKD following LT are shown in Table 3. Age (*P* =.019) and MAKE30 (*P* =.046) were the independent factors associated with CKD onset. To explore the impact of these significant CKD determinants, we constructed an ROC curve using age as a parameter (Figure S1). The corresponding AUC was 0.599, with an optimal age cut-off of 49 years. A detailed performance metric for age in predicting the incidence of CKD is presented in Supplementary Table 1. Patients were dichotomized at 49 years of age, resulting in a sensitivity of 52.8% and a specificity of 66.7%. Figure 2 illustrates the risk of CKD progression when age was combined with the occurrence of L-AKI or with the occurrence of MAKE30. In the cohort of individuals aged ≤ 49 years without L-AKI, only 23% developed CKD, whereas all 10 patients aged > 49 years who experienced L-AKI developed CKD (Fig. 2A). Similarly, among individuals aged ≤ 49 years without MAKE30, only 27% developed CKD, while all 4 patients aged > 49 years who met the MAKE30 criteria developed CKD (Fig. 2B).

**Table 3:** Multivariate analyses of chronic kidney disease following lung transplantation

Variables	Hazard ratio (95% CI)	*P* value
Age	1.065 (1.011–1.122)	0.019
Preoperative Hb	0.896 (0.665–1.206)	0.468
Preoperative albumin	0.485 (0.138–1.706)	0.303
Preoperative ECMO	>100 (0.00 -)	0.999
Intraoperative circulatory support time	0.999 (0.994–1.004)	0.657
Operation time	1.002 (0.999–1.006)	0.241
Postoperative RRT	1.636 (0.083–32.483)	0.747
Postoperative L-AKI	2.190 (0.483–9.935)	0.309
MAKE30	9.821 (1.045–92.321)	0.046

*CI* confidence interval; *Hb* hemoglobin; *ECMO* extracorporeal membrane oxygenation; *RRT* renal replacement therapy; *L-AKI* late acute kidney injury; *MAKE30* Major Adverse Kidney Events within 30 days after lung transplant

**Fig. 3:**
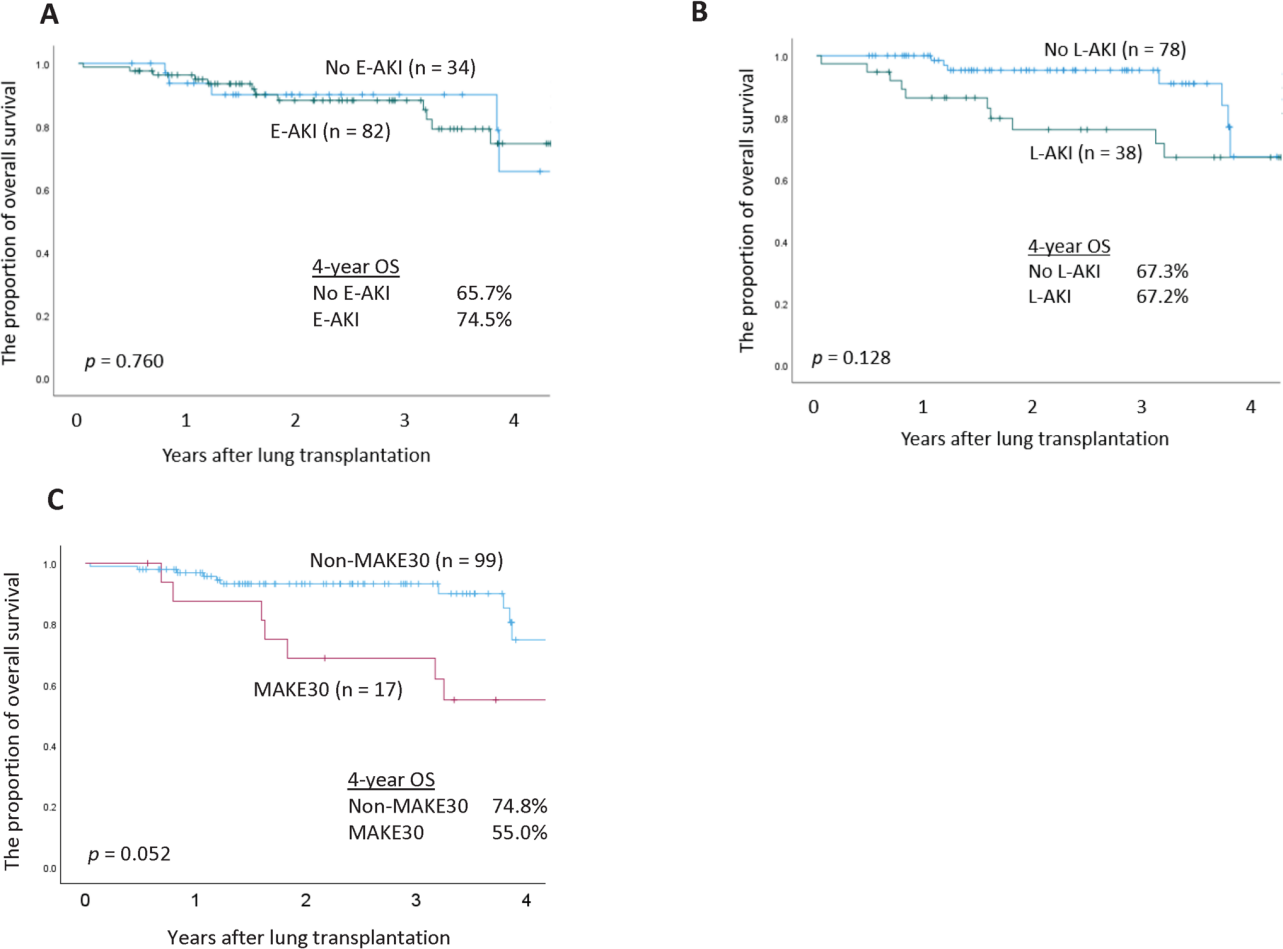
Overall survival (OS) curves of patients undergoing lung transplantation. **A** OS curves according to the presence or absence of early acute kidney injury (E-AKI). **B** OS curves according to the presence or absence of late acute kidney injury (L-AKI). **C** OS curves according to the presence or absence of Major Adverse Kidney Event within 30 days

Kaplan–Meier curves demonstrated that the presence of E-AKI was not significantly associated with overall survival (OS) (*P* =.760; Fig. 3A). Although there was a trend suggesting a potential association between L-AKI and OS, the result did not reach statistical significance (*P* =.128; Fig. 3B). Similarly, MAKE30 showed a marginal association with OS (*P* =.052; Fig. 3C).

## Discussion

The prevalence of CKD after LT is high, ranging from 15.8% to 68.6% among LT recipients [5, 12]. However, the reported numbers vary, owing to differences in the definitions of CKD. We adopted the criteria for postoperative CKD, which defines it as patients having an eGFR < 60 mL/min/1.73m^2^ persisting for more than 3 months post-LT. These criteria are frequently used to diagnose chronic renal failure. The incidence of CKD (46%) found is consistent with that of earlier studies. The need for dialysis shortly after LT escalates the adjusted hazard ratio for 1-year and 5-year mortality to 7.2 and 4.0, respectively [2]. Thus, determining the causal factors of CKD is crucial although we did not assess OS based on the presence of CKD owing to the limited follow-up duration. Numerous factors have been identified as contributors to the onset of post-LT CKD. These include use of immuno-suppressants, occurrences of AKI, high lung allocation scores, advanced age, male sex, pre-existing low eGFR, increased albuminuria, diabetes mellitus, pulmonary hypertension, and heart failure, among other factors [1, 3–10, 17, 19–21]. Nevertheless, age and MAKE30 emerged as significant predictors of CKD in our study. The combination of age ≥49 years and MAKE30 had a notable impact on predicting the development of CKD. However, age alone may have limited utility as a predictive marker, given the relatively low performance metrics, including a modest AUC.

Kidney function deteriorates with age, making older recipients more susceptible to AKI and its subsequent progression to CKD. Factors, such as the use of nephrotoxic agents and instability in hemodynamics, intensify the risk of rapid impairment. With the forecasted increase in older recipients, there is a need to amplify renal care and fluid balance during the acute and sub-acute phases post-LT. This is pivotal for curtailing the progression of CKD.

Prolonged use of calcineurin inhibitors is a recognized nephrotoxic agent, because of its association with acute arteriolopathy [18, 21, 22]. A randomized trial demonstrated the efficacy of everolimus in preserving renal function when used in conjunction with reduced calcineurin inhibitor regimens although this regimen is not covered by public health insurance in Japan [20]. We evaluated the variations in absolute tacrolimus blood levels using CV, but it did not indicate a relationship with CKD. One possible reason is the limited number of recipients in the sample. The CV of tacrolimus levels has been linked to acute rejection and donor-specific antibodies [18, 21, 22]. A study assessing the correlation between survival and CKD in a larger recipient pool would offer insight into the mechanism of CKD progression.

AKI is recognized as a precursor to irreversible renal impairment after LT [8, 9]. In our study, E-AKI was more prevalent than L-AKI, with incidence rates of 73% and 31%, respectively. Patients are vulnerable to hemodynamic instability in the immediate postoperative period. Considerable stress can easily disrupt fluid homeostasis, triggering AKI. This is one of the main reasons for the discrepancy between the incidences of E-AKI and L-AKI. The connection between E-AKI and CKD is often observed as a phase in a continuous spectrum of the same component. The term L-AKI indicates the transition from AKI to CKD, representing an interval in which timely intervention and risk management could alter this adverse progression. Managing the transitional period during which L-AKI emerges is instrumental in preventing CKD progression.

Many studies have underscored the significance of post-LT AKI on mortality, revealing a pooled risk of in-hospital mortality for patients with AKI and severe AKI requiring renal replacement therapy at 2.75 and 10.89, respectively [9]. MAKE30 is a patient-centered indicator that reflects the short-term clinical impact of AKI [13, 15]. This metric helps clinicians identify high-risk patients early, decide on the appropriate treatment strategies, and monitor patient recovery during the immediate postoperative period. Previous research has shown that MAKE30 can be easily extracted from electronic medical records, which supports its use in daily clinical practice as well as in clinical trials [14, 15]. Our findings indicate that MAKE30 has the strongest prognostic value and is independently associated with the development of CKD. Given the predictive significance of both MAKE30 and L-AKI for CKD, we propose that extended follow-up for AKI diagnosis is essential to evaluate postoperative factors, such as exposure to nephrotoxic agents, sepsis, and other complications. These findings support the need for ongoing AKI assessment for up to one month following LT, emphasizing the importance of rigorous postoperative care to prevent renal damage during the sub-acute period. For risk stratification and early identification, the components of MAKE30 should be integrated into routine post-AKI assessments to identify high-risk patients. Furthermore, clinical scoring systems or electronic alert tools can be utilized to flag patients who meet MAKE30 criteria. For post-AKI monitoring, structured follow-up should be implemented for patients who experience MAKE30 events, ideally within 30 to 90 days post-discharge. Monitoring of renal function trends—such as eGFR, serum creatinine, and albuminuria—is essential to detect early CKD progression.

This study had several limitations. First, it was a single-center, retrospective study with a relatively limited sample size, which may reduce the statistical power and limit the generalizability of the findings to broader populations or different clinical settings. Second, due to the retrospective design, potential confounding factors could not be fully controlled, and causal relationships could not be established. Third, because of data constraints, a detailed evaluation of the dosage, duration, and timing of exposure to nephrotoxic agents was not feasible.

Future research should include prospective, multicenter studies with larger cohorts to validate these findings and improve generalizability. Additionally, randomized controlled trials are warranted to explore the causal impact of MAKE30 and L-AKI on CKD progression, as well as to evaluate the efficacy of targeted interventions in high-risk patients. In conclusion, post-LT emergence of L-AKI and MAKE30 plays a significant role in the development of CKD and is associated with poorer long-term survival. MAKE30 is simple but independently associate with CKD. These findings highlight the importance of optimal postoperative care to mitigate kidney damage beyond the first week after LT, which is especially critical for older recipients to curb CKD progression.

## Supplementary Information

Below is the link to the electronic supplementary material.

Supplementary file1 (DOCX 12 kb)

Supplementary file2 (JPG 193 kb)

Supplementary file3 (DOCX 14 kb)

## Data Availability

The data underlying this article cannot be shared publicly due to privacy or ethical restrictions.

## Abbreviations

AKI: Acute kidney injury
AUC: Area under the curve
CKD: Chronic kidney disease
CPB: Cardiopulmonary bypass
CV: Coefficient of variation
E-AKI: Early-acute kidney injury
ECMO: Extracorporeal membrane oxygenation
eGFR: Estimated glomerular filtration rate
KDIGO: Kidney disease improving global outcomes
L-AKI: Late-acute kidney injury
LT: Lung transplantation
MAKE 30: Major adverse kidney event within 30 days
OS: Overall survival
ROC: Receiver operating characteristics
sCr: Serum creatinine
