# Supplementary material for: Early major adverse kidney events after lung transplantation: risk of chronic kidney disease and prognostic impact
Source: Gen Thorac Cardiovasc Surg. 2025 Aug 28;74(2):203–10. doi: 10.1007/s11748-025-02193-4 (PMC12913340; doi:10.1007/s11748-025-02193-4)
Supplement: Supplementary file 1 — Supplementary file1 (DOCX 12 kb) [file 11748_2025_2193_MOESM1_ESM.docx]

Supplementary Figure 1. Receiver-operating characteristics area under the curve (AUC) (0.599, 95% confidence interval [CI], 0.496 to 0.703, P = .061) for age used to identify the incidence of chronic renal disease after lung transplantation.
