# Supplementary figures and images for: Early major adverse kidney events after lung transplantation: risk of chronic kidney disease and prognostic impact
Source: Gen Thorac Cardiovasc Surg. 2025 Aug 28;74(2):203–10. doi: 10.1007/s11748-025-02193-4 (PMC12913340; doi:10.1007/s11748-025-02193-4)

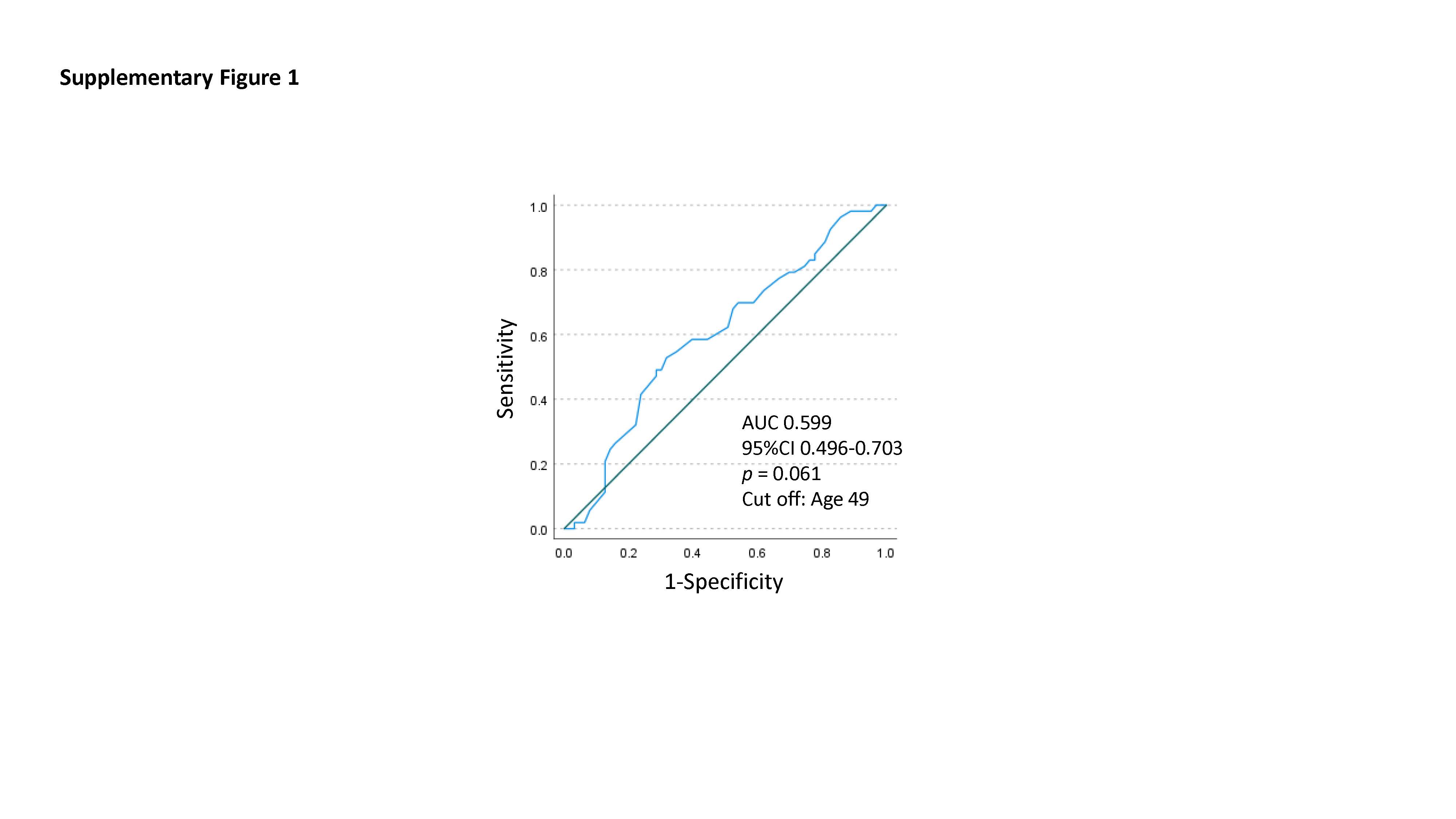

Supplement: Supplementary file 2 — Supplementary file2 (JPG 193 kb) [file 11748_2025_2193_MOESM2_ESM.jpg]
