# Supplementary material for: Early major adverse kidney events after lung transplantation: risk of chronic kidney disease and prognostic impact
Source: Gen Thorac Cardiovasc Surg. 2025 Aug 28;74(2):203–10. doi: 10.1007/s11748-025-02193-4 (PMC12913340; doi:10.1007/s11748-025-02193-4)
Supplement: Supplementary file 3 — Supplementary file3 (DOCX 14 kb) [file 11748_2025_2193_MOESM3_ESM.docx]

Supplementary table 1: The performance of age in predicting the incidence of chronic kidney disease after post-lung transplant

| Chronic kidney disease | Age | | AUC | Sensitivity (%) | Specificity (%) |
| --- | --- | --- | --- | --- | --- |
|  | < 49 | > 49 |  |  |  |
| Negative  Positive | 42 | 21 | 0.599 | 52.8 | 66.7 |
|  | 25 | 28 |  |  |  |
